# Folic Acid Reinforces Maize Tolerance to Sodic-Alkaline Stress through Modulation of Growth, Biochemical and Molecular Mechanisms

**DOI:** 10.3390/life12091327

**Published:** 2022-08-27

**Authors:** Bandar S. Aljuaid, Soumya Mukherjee, Amany N. Sayed, Yasser Abd El-Gawad El-Gabry, Mohamed M. A. Omar, Samy F. Mahmoud, Moodi Saham Alsubeie, Doaa Bahaa Eldin Darwish, Salem Mesfir Al-Qahtani, Nadi Awad Al-Harbi, Fahad Mohammed Alzuaibr, Mohammed A. Basahi, Maha M. A. Hamada

**Affiliations:** 1 Department of Biotechnology, College of Science, Taif University, P.O. Box 11099, Taif 21944, Saudi Arabia; 2 Department of Botany, Jangipur College, University of Kalyani, Kalyani 742213, India; 3 Department of Agronomy, Faculty of Agriculture, Ain Shams University, Cairo 11566, Egypt; 4 Department of Biochemistry, Faculty of Agriculture, Ain Shams University, Cairo 11566, Egypt; 5 Biology Department, College of Science, Imam Mohammad Ibn Saud Islamic University (IMSIU), Riyadh 11623, Saudi Arabia; 6 Botany Department, Faculty of Science, Mansoura University, Mansoura 35511, Egypt; 7 Biology Department, Faculty of Science, Tabuk University, Tabuk 71491, Saudi Arabia; 8 Biology Department, University College of Tayma, University of Tabuk, P.O. Box 741, Tabuk 47512, Saudi Arabia; 9 Department of Biology, Faculty of Science, University of Tabuk, P.O. Box 741, Tabuk 71491, Saudi Arabia; 10 College of Science and Arts Sajir, Shaqra University, P.O. Box 33, Shaqra 11961, Saudi Arabia

**Keywords:** folates, *Zea maize*, ion homeostasis, sodium efflux, alkalinity, leaf pigments and osmolytes

## Abstract

The mechanism by which folic acid (FA) or its derivatives (folates) mediates plant tolerance to sodic-alkaline stress has not been clarified in previous literature. To apply sodic-alkaline stress, maize seedlings were irrigated with 50 mM of a combined solution (1:1) of sodic-alkaline salts (NaHCO_3_ and Na_2_CO_3_; pH 9.7). Maize seedlings under stressed and non-stressed conditions were sprayed with folic acid (FA) at 0 (distilled water as control), 0.05, 0.1, and 0.2 mM. Under sodic-alkaline stress, FA applied at 0.2 mM significantly improved shoot fresh weight (95%), chlorophyll (Chl a (41%), Chl b (57%), and total Chl (42%)), and carotenoids (27%) compared to the untreated plants, while root fresh weight was not affected compared to the untreated plants. This improvement was associated with a significant enhancement in the cell-membrane stability index (CMSI), relative water content (RWC), free amino acids (FAA), proline, soluble sugars, K, and Ca. In contrast, Na, Na/K ratio, H_2_O_2_, malondialdehyde (MDA), and methylglycoxal (MG) were significantly decreased. Moreover, seedlings treated with FA demonstrated significantly higher activities of antioxidant enzymes including superoxide dismutase (SOD), peroxidase (POX), catalase (CAT), and ascorbate peroxidase (APX) compared to the untreated plants. The molecular studies using RT-qPCR demonstrated that FA treatments, specifically at 0.2 mM, enhanced the K^+^/Na^+^ selectivity and the performance of photosynthesis under alkaline-stress conditions. These responses were observed through up-regulation of the expression of the high-affinity potassium-transporter protein (*ZmHKT1*), the major core protein of photosystem II (*D2-Protein*), and the activity of the first enzyme of carbon fixation cycle in C4 plants (*PEP-case*) by 74, 248, and 225% over the untreated plants, respectively. Conversely, there was a significant down-regulation in the expression *ZmSOS1* and *ZmNHX1* by 48.2 and 27.8%, respectively, compared to the untreated plants.

## 1. Introduction

Salt-affected soils have become a major global concern due to increasing population and food demand [1]. These soils have been estimated to cover 1125 million hectares and, due to human activities, are expected to increase annually [2]. When plants are subjected to saline conditions, they produce excessive levels of reactive oxygen species (ROS) such as superoxide anion (O^−2^), hydroxyl radical (HO•), and hydrogen peroxide (H_2_O_2_) [3]. This overproduction of ROS causes lipid peroxidation and metabolic disorders, and even leads to programmed cell death [4].

Sodic-alkaline stress is considered one of the greatest environmental threats to the agricultural sector and the achievement food security worldwide. It causes two risk factors at the same time: salinity and alkalinity [5,6], and can negatively affect respiratory metabolism, antioxidant systems, nutritional status, and organic acid metabolism [2]. Furthermore, sodic-alkaline stress has harmful impacts on the cell-membrane stability index, plant–water relations, photosynthetic pigments, and a wide array of physiological, biochemical, and molecular aspects [5,6,7]. When plants are grown under saline or alkaline conditions, they are subjected to three major problems: (i) water deficiency through osmotic stress and the hindering water uptake, (ii) disturbance of the plant nutritional status through affecting ion-transport systems, and (iii) sodium toxicity by increasing its uptake in the susceptible tissues [2,6,7,8,9,10]. Exposing plants to saline and/or alkaline stress can affect the concentration of osmolytes (sugars, amino acids, polyamines, etc.) and trigger an intracellular-signaling cascade including the generation of secondary messenger molecules such as Ca^2+^ and protons [10,11,12]. Furthermore, alkaline stress can affect the structural organization of the genetic apparatus in plants by disturbance of the chromatin compaction and occurrence of nuclear bodies of unknown etiology in the apical meristem [13].

Folic acid and its derivatives (folates) are known as the water-soluble vitamin B9 that ubiquitous synthesized in the plant kingdom [14]. They are involved in the linking between the metabolisms of carbon and nitrogen in plants [15]. Furthermore, folates play a major role in DNA synthesis and one-carbon transfer reactions [15,16]. When deficient in folates, plants are unable to grew normally due to reducing the genome stability and affecting the rate of cell division [14]. It has been found that plants exposed to adverse conditions are more susceptible to folate deficiency due to the quick decline in the regulation of folate biosynthesis genes [17,18,19]. In addition, folates are involved in the biosynthesis of various amino acids, phytohormones, photosynthetic pigments, and the lipid protective antioxidant vitamin E [15]. These effects can be explained by the folates’ ability to stimulate many precursors and intermediate pathways such as the biosynthesis of porphyrins, S-adenosylmethionine (SAM), and isoprenoids [15,20]. On the other hand, exogenous folic acid has demonstrated strong antioxidant properties that are responsible for reducing the oxidative damage in several plant species under various abiotic stresses, i.e., drought [20,21], salinity [8,22,23], and heavy metals [24]. Generally, when abiotic-stressed plants are treated with a strong antioxidant, other toxic molecule such as methylglyoxal (MG) can be diminished [25]. This strategy is extremely important in the regulation of plant-signaling processes, growth and development, and various metabolic pathways under stress conditions [26,27].

Maize (*Zea mays*) is one of the most important cereal crops cultivated worldwide [28,29]. It provides suitable raw materials for several industries including biofuels, fodder, silage, and starch in addition to its high nutritional value for humans and animals [30,31,32]. Under saline conditions, maize demonstrates severe damage and significant losses in its growth and productivity [33]. Furthermore, alkaline stress can be more dangerous than salinity alone in its effect on growth and different metabolic processes [7]. Therefore, sodic-alkaline stress has become a major global concern because it can greatly restrict the cultivation of maize in many regions of the world.

Until now, there has been no conclusive data on the effect of folic acid on maize plants under sodic-alkaline stress. Therefore, this study was conducted to investigate the possible protective influences of folic acid as a foliar application on several growth, biochemical, and molecular aspects related to plant tolerance to sodic-alkaline stress.

## 2. Materials and Methods

### 2.1. Plant Material and Growth Conditions and Treatments

Maize grains of the white single-cross hybrid (Hytech 2030) produced by Misr Hytech Seed Int., Nasr City, Egypt were sterilized by soaking in 0.5% NaOCl for 5 min and washed with distilled water four times. Grains were germinated on wetted filter paper at 25 °C for 24 h. Seedlings with uniform size were selected and transferred into black plastic pots (20 cm diameter) filled with 8 kg pre-washed sand (five seedlings/pot). All pots were placed in plant growth chamber (28/18 °C day/night; 14/10 h light/dark; 180 µmol m^−2^ S^−1^ light intensity, 70% relative humidity) and irrigated with half strength Hoagland’s solution every two days. After two weeks, all pots were divided into two major groups of sodic-alkaline stressed and unstressed conditions. To apply the sodic-alkaline stress, seedlings of the stressed group were irrigated with a modified half-strength Hoagland’s nutrient solution containing two alkaline salts (NaHCO_3_ and Na_2_CO_3_) that were mixed in a ratio 1:1 (50 mM; pH 9.7). Meanwhile, those of the unstressed group were irrigated with half-strength Hoagland’s nutrient solution.

### 2.2. Foliar Applications of Folic Acid and the Experimental Layout

Pots in each major group were divided into four subgroups to apply the folic acid treatments (FA; Oxford Laboratory Reagent Company; Maharashtra, India). Each subgroup was sprayed with one of the following solutions: (1) distilled water under non-stressed conditions, (2) 0.05 mM FA under non-stressed conditions, (3) 0.1 mM FA under non-stressed conditions, (4) 0.2 mM FA under non-stressed conditions, (5) distilled water under sodic-alkaline conditions, (6) 0.05 mM FA under sodic-alkaline conditions, (7) 0.1 mM FA under sodic-alkaline conditions, and (8) 0.2 mM FA under sodic-alkaline conditions. The volume of spraying solution was 25 mL/pot and repeated five times at 15, 17,19, 21, and 24 days after transplanting. All foliar treatments were applied with 0.05% (V/V) Tween-20 as a wetting agent. The samples were collected after a week to estimate the different studied traits. The experimental layout was a completely randomized design (CRD) with three replicates. The total number of pots was 120 (2 alkaline treatments × 4 foliar concentrations × 5 pots × 3 replicates).

### 2.3. Growth Parameters and Life Pigments

After gathering of seedlings at 31 days after transplanting, shoot and root fresh weights were determined immediately using a digital balance. Chl a, Chl b, and carotenoids were determined as described by Lichtenthaler and Wellburn [34].

### 2.4. Cell Membrane Integrity and Oxidative Damage

The cell-membrane stability index was estimated as described by Abd Elbar, et al. [35]. Some leaf discs (8) were incubated for 24 h in 10 mL deionized water on a shaker. Then *EC*_1_ values of contents were measured by *EC* meters. Samples were autoclaved at 120 °C for 20 min to determine the values of *EC*_2_. The cell-membrane stability index was calculated using the following equation:

$$MSI=\left[1-\left(\frac{EC1}{EC2}\right)\right]\times 100$$

Methylglyoxal (MG) content was determined using a UV-spectrophotometer at 335 nm according to Hossain, Hossain, and Fujita [26]. Hydrogen peroxide (H_2_O_2_) concentration was estimated colorimetrically by the potassium iodide method [36]. Malondialdehyde (MDA) was determined using the thiobarbituric acid method (TBA) [37].

### 2.5. Determination of Total Soluble Protein and Activates of Antioxidant Enzymes

Total soluble protein was determined in the enzyme extract according to Bradford [38]. Ascorbate peroxidase (APX; EC 1.11.1.11) activity was determined based on the decrease in ascorbate at 290 nm [39]. Catalase (CAT; EC 1.11.1.6) activity was assayed by monitoring the decrease in absorbance of H_2_O_2_ at 240 nm [40]. Guaiacol peroxidase (G-POX; EC1.11.1.7) activity was evaluated by observing its ability to convert guaiacol to tetraguaiacol by monitoring the increase in absorbance at 470 nm [41]. Superoxide dismutase (SOD; EC 1.15.1.1) activity was evaluated according to the ability to inhibit the photochemical reduction of nitro blue tetrazolium (NBT) at 560 nm [42].

### 2.6. Determination of Leaf Relative Water Content and Osmotic Compounds

The method of Abd El-Gawad, et al. [43] was used to determine the leaf relative water content (RWC).

Total soluble sugars were determined as described by Chow and Landhäusser [44]. Free amino acids were determined by ninhydrin reagent as glycine according to the method of Hamilton, et al. [45]. Proline concentration was estimated according to Bates, et al. [46].

### 2.7. Determination of Na, K, and Ca

Leaf mineral concentrations of Na, K, and Ca were determined using the flame photometric method (Jenway, Leicestershire, UK) as described by Havre [47].

### 2.8. Gene Expression

The total mRNA from different treatments was extracted according to the manufacturer’s protocol, using 0.5 g of fresh leaves using an RNA extraction kit (Sigma-Aldrich, St. Louis, MO, USA). The quality of purified RNA was quantitated using NanoDrop™2000/2000c Spectrophotometers, after the reverse transcription of RNA and cDNA formation according to the manufacturer’s protocol (Promega, Walldorf, Germany). Real-time quantitative reverse-transcription polymerase chain reaction (qRT-PCR) analysis (Rotor-Gene 6000, Hilden, Germany) was performed using real-time PCR on 1 L diluted cDNA in triplicate and the primer sequences used in qRT-PCR are provided in Table 1. The β-Actin housekeeping gene (reference gene) was utilized to analyze gene expression using SYBR^®^ Green. The relative gene expression was determined using the 2ΔDDCt method [48].

### 2.9. Statistical Analysis

All data were subjected to one-way ANOVA; while the differences between means ± SE from three replicates were determined using Tukey’s multiple range test at *p* ≤ 0.05 using SAS [49].

## 3. Results

### 3.1. Effect of Folic Acid on Plant Growth and Photosynthetic Pigments

**Figure 1:**
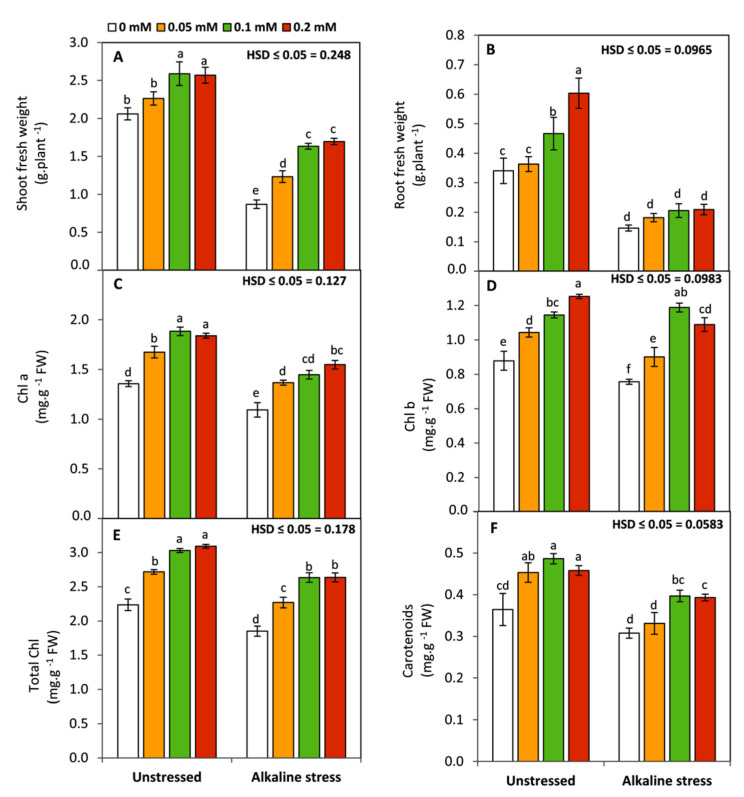
Effect of variable concentrations of folic acid (0.05–0.2 mM) on shoot fresh weight (**A**), root fresh weight (**B**), chl content (**C**–**E**), and carotenoid content (**F**) of leaves of 31-day-old maize seedlings raised in the absence and presence of sodic-alkaline stress. Values (n = 6 ± SE) with different letters are significantly different at *p* < 0.05 according to Tukey’s multiple range.

Maize seedlings exposed to sodic-alkaline stress showed a significant (*p* ≤ 0.05) decrease in shoot and root fresh weight, Chl a, Chl b, and total Chl compared to the unstressed plants. Meanwhile, carotenoids were not affected (Figure 1). Folic acid applications specifically at 0.1 and 0.2 mM to non-alkaline or alkaline-stressed plants displayed a significant improvement in shoot fresh-weight, Chl a, Chl b, total Chl, and carotenoids compared to the untreated plants. However, this effect was observed in root fresh-weight under non-alkaline-stress conditions only.

### 3.2. Effect of Folic Acid on Membrane Stability Index and Oxidative-Damage Markers

**Figure 2:**
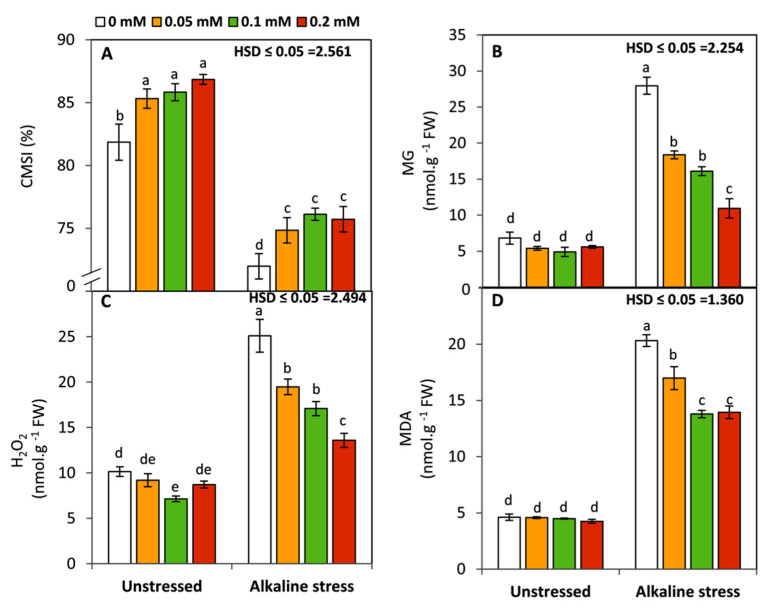
Effect of variable concentrations of folic acid (0.05–0.2 mM) on CMSI (%) (**A**), methylglyoxal content (**B**), hydrogen peroxide content (**C**), and malondialdehyde content (**D**) of leaves of 31-day-old maize seedlings raised in the absence and presence of sodic-alkaline stress. Values (n = 6 ± SE) with different letters are significantly different at *p* < 0.05 according to Tukey’s multiple range.

Under sodic-alkaline stress, an obvious and significant (*p* ≤ 0.05) decrease in CMSI was observed with increasing the oxidative damage as indicated by increasing the level of MG, H_2_O_2_, and MDA in the stressed seedling (Figure 2). Conversely, applied-FA significantly (*p* ≤ 0.05) enhanced CMSI under non-alkaline and alkaline stressed conditions compared with the untreated plant. Moreover, there was an obvious and significant (*p* ≤ 0.05) decrease in MG, H_2_O_2_, and MDA associated with increasing the concentration of FA up to 0.2 mM. These results imply that FA treatments can maintain the structure and functions of cell membranes by reducing the oxidative damage.

### 3.3. Effect of Folic Acid on the Activities of Antioxidant Enzymes

**Figure 3:**
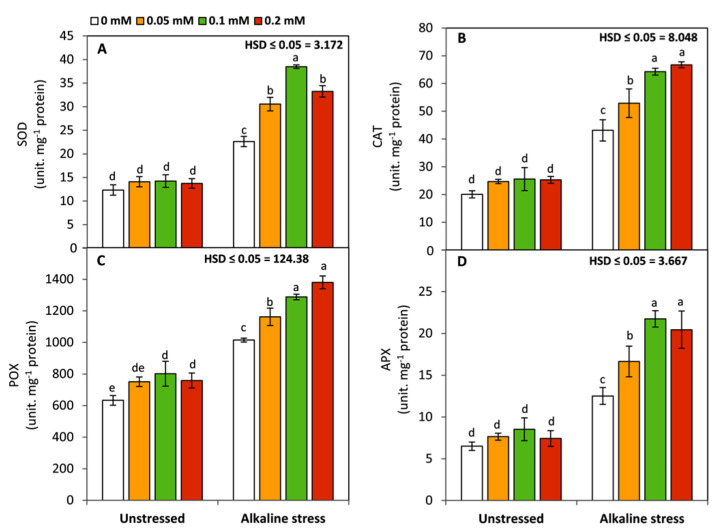
Effect of variable concentrations of folic acid (0.05–0.2 mM) on the activity of SOD (**A**), CAT (**B**), POX (**C**), and APX (**D**) in leaves of 31-day-old maize seedlings raised in the absence and presence of sodic-alkaline stress. Values (n = 6 ± SE) with different letters are significantly different at *p* < 0.05 according to Tukey’s multiple range.

Under non sodic-alkaline conditions, no changes were detected in the activities of SOD, CAT, or APX between FA-treated and untreated seedlings. However, POX exhibited an obvious and significant (*p* ≤ 0.05) increase with FA treatment at 0.1 and 0.2 mM (Figure 3). Conversely, all examined antioxidant enzymes including SOD, CAT, POX, and APX were significantly (*p* ≤ 0.05) increased by the different FA treatments. Generally, the most significant (*p* ≤ 0.05) findings were achieved by FA treatment at 0.1 or 0.2 mM.

### 3.4. Effect of Folic Acid on Leaf Relative Water Content and Osmolytes

**Figure 4:**
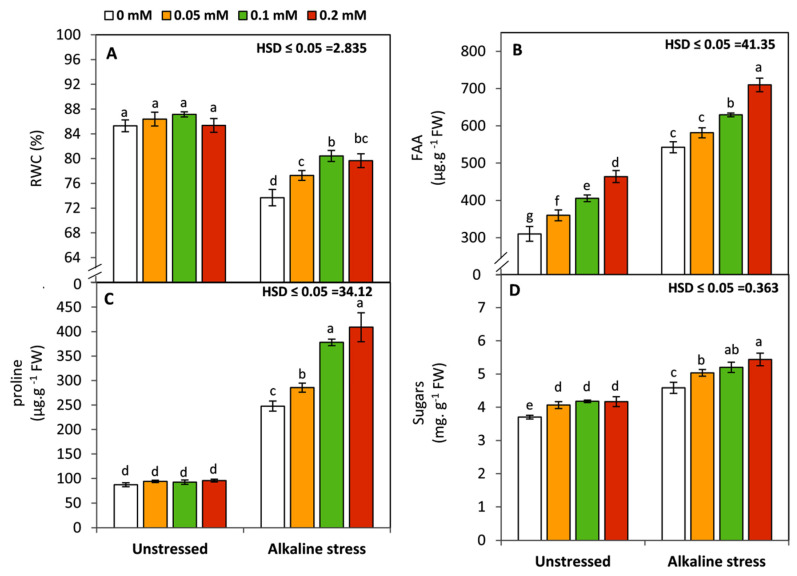
Effect of variable concentrations of folic acid (0.05–0.2 mM) on RWC (**A**), FAA content (**B**), proline content (**C**), and sugar content (**D**) of leaves of 31-day-old maize seedlings raised in the absence and presence of sodic-alkaline stress. Values (n = 6 ± SE) with different letters are significantly different at *p* < 0.05 according to Tukey’s multiple range.

Maize seedlings exposed to sodic-alkaline stress displayed an obvious and significant (*p* ≤ 0.05) decrease in RWC compared to those in unstressed conditions. Conversely, an opposite trend was observed in FAA, proline, and soluble sugars (Figure 4). Generally, under non sodic-alkaline conditions, seedlings treated by FA showed significant (*p* ≤ 0.05) increase in FAA and soluble sugars compared to the untreated plants, while, no significant changes were detected in RWC and proline. However, under sodic-alkaline conditions, RWC, FAA, proline, and soluble sugars were significantly (*p* ≤ 0.05) enhanced by FA treatments compared to the untreated seedlings. This improvement was in parallel with increasing the FA concentration.

### 3.5. Effect of Folic Acid on Na, K, and Ca Concentration

**Figure 5:**
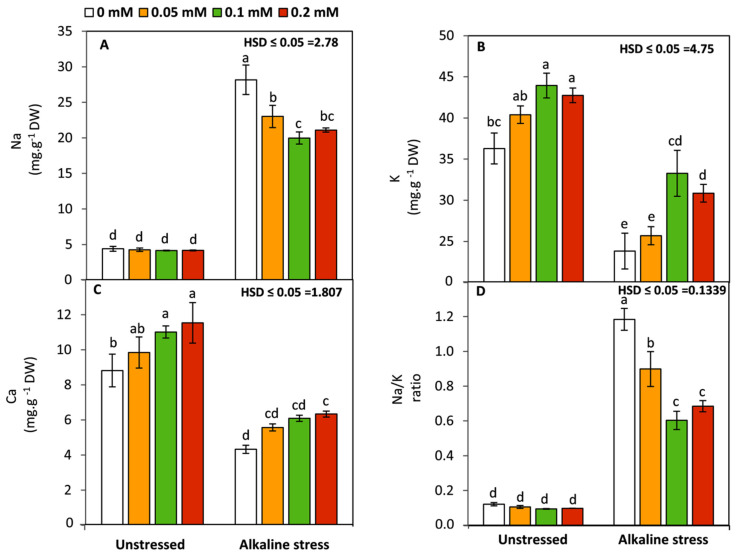
Effect of variable concentrations of folic acid (0.05–0.2 mM) on Na^+^ (**A**), K^+^ (**B**), Ca^2+^ content (**C**), and Na^+^/K^+^ ratio (**D**) of leaves of 31-day-old maize seedlings raised in the absence and presence of sodic-alkaline stress. Values (n = 6 ± SE) with different letters are significantly different at *p* < 0.05 according to Tukey’s multiple range.

Sodic-alkaline stress led to a significant (*p* ≤ 0.05) increase in Na, while, K and Ca were significantly decreased compared to the unstressed conditions (Figure 5). These influences dramatically and negatively affected Na/K ratio in the leaf tissues of sodic-alkaline-stressed seedlings compared to those of unstressed conditions. Applied-FA at 0.1 and 0.2 mM significantly (*p* ≤ 0.05) enhanced K and Ca under non-sodic-alkaline conditions, but did not affect Na or the Na/K ratio. In contrast, it was observed that applied FA led to a substantial increase in K and Ca associated with a significant (*p* ≤ 0.05) decrease in the Na and Na/K ratios compared to the untreated seedlings. In this context, the treatments of FA at 0.1 and 0.2 mM were more potent than those at the lower concentration of 0.05 mM.

### 3.6. Effect of Folic Acid on the Expression of Photosynthesis and Salt-Stress-Responsive Genes

**Figure 6:**
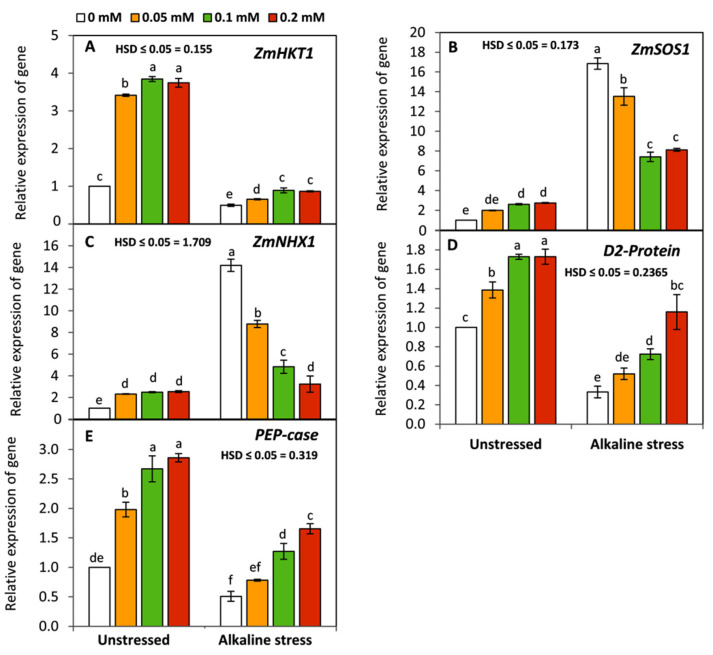
Effect of variable concentrations of folic acid (0.05–0.2 mM) on gene expression of *ZmHKT1* (**A**), *ZmSOS1* (**B**), *ZmNHX1* (**C**) *D_2_ protein* (**D**), and *PEP-case* (**E**) of leaves of 31-day-old maize seedlings raised in the absence and presence of sodic-alkaline stress. Values (n = 3 ± SE) with different letters are significantly different at *p* < 0.05 according to Tukey’s multiple range.

As shown in Figure 6, regardless of the presence of FA, sodic alkaline stress significantly (*p* ≤ 0.05) down-regulated the relative expression of *ZmHKT1*, *D2-Protein,* and *PEP-case.* Meanwhile, *ZmSOS1* and *ZmNHX1* were up-regulated. Conversely, under sodic-alkaline stress, an obvious and significant (*p* ≤ 0.05) decrease in the expression of *ZmSOS1* and *ZmNHX1* was observed in FA-treated seedlings. Furthermore, applied-FA significantly up-regulated the expression of *ZmHKT1*, *D2-Protein,* and *PEP-case* compared to the untreated seedlings. These results imply that FA can improve plant tolerance to sodic-alkaline stress by regulating the uptake of K and Na on one hand, and enhancing the photosynthetic capacity on the other.

## 4. Discussion

In this study, plants exposed to sodic-alkaline stress displayed a significant inhibition in plant growth as indicated by reduced shoot and root fresh weights compared to those in unstressed conditions. Generally, salinity can seriously restrict plant growth and development in many plant species due to the ionic toxicity and osmotic stress [8,50,51]. Moreover salt stress suppresses the rate of cell division [52], homeostasis of phytohormones [53], capacity of photosynthesis [54], and uptake of nutrients [2,55]. On the other hand, alkaline stress can cause more damage to plants than salt stress alone due to the presence of high pH in addition to the toxic effect of sodium ions [6,7,12]. In contrast, it was observed that plants treated with FA showed significant enhancement in growth parameters under unstressed and sodic-alkaline conditions. These effects could be attributed to the positive effect of FA on cell division, genome stability, and gene expression [14,56,57] reducing the cytotoxic effects of salinity stress [58]. Furthermore, FA and its derivatives (folates) are implicated in the synthesis of a wide array of amino acids including methionine, glycine, tryptophan, glutamic acid and valine that may be involved in the biosynthetic pathways of multiple plant growth regulators such as auxins, polyamines, and ethylene [15].

Photosynthetic pigments (Chl a, Chl b, and Chl a+b) were also shown to be negatively and significantly affected by sodic-alkaline stress, but carotenoids were not changed. Diminishing of chlorophyll content under various abiotic-stress conditions can be considered an important regulatory step to avoid the over-reduction of the photosynthetic electron transport chain and consequently restrict the excessive generation of ROS [59]. Meanwhile, the stability of carotenoids as efficient non-enzymatic antioxidants can play a protective role for the photosynthetic apparatus, reducing oxidative damage under sodic-alkaline stress. In addition, FA treatments especially at 0.1 and 0.2 mM significantly enhanced the content of chlorophylls and carotenoids. Generally, folates can be indirectly involved in the biosynthesis of porphyrins and S-adenosylmethionine leading to the formation of chlorophylls and all isoprenoids such as carotenoids and α-tocopherol (vitamin E), respectively [15].

Under sodic-alkaline stress, an obvious and significant decrease in CMSI was observed with increasing the oxidative damage, as indicated by the elevated level of MG, H_2_O_2_, and MDA in the stressed seedling. Several lines of evidence indicated that under abiotic-stress conditions, plants trigger a number of biochemical markers which function as signaling molecules to evolve the defensive mechanisms against this stress [3,60,61]. However, the excessive accumulation of these molecules, including reactive oxygen species (ROS) such as hydroxyl radicals (OH), alkoxy radicals (RO), superoxide anion radicals (O^−^_2_), singlet oxygen (O^1^_2_), and hydrogen peroxide (H_2_O_2_) can cause serious degeneration to various plant tissues and physiological processes [10,35,62,63,64]. Furthermore, methylglycoxal (MG) as a reactive carbonyl species has been found to be toxic at high levels leading to the restriction of plant growth and development by affecting photosynthesis, stomatal movement, cytosolic calcium, root differentiation, and seed germination [25,65,66]. Together these molecules can affect the function and stability of cell membranes by elevating the rate of lipid peroxidation (raising the level of malondialdehyde; MDA), in addition to causing severe degenerative oxidative stress to proteins. In this study, seedlings treated with FA exhibited an obvious improvement in CMSI and a reduction in the biochemical markers of carbonyl and oxidative stress under sodic-alkaline stress. Exogenous FA has been found to play a crucial role in reducing oxidative damage under abiotic-stress conditions such as irrigation deficit [20,21] and salinity [8,23]. These properties might be due to its ability to enhance the glutathione–ascorbate cycle [14]. Moreover FA and its derivatives are involved in the biosynthesis of important non-enzymatic antioxidants such as α-tocopherol and carotenoids via stimulation of the isoprenoid pathway [15]. In this context, FA has been shown to improve the integrity of cell membranes by diminishing lipid peroxidation [67,68].

Increase in the activities of antioxidant enzymes is considered a common response in several plant species under various abiotic-stress conditions [9,69,70]. These effects enable plants to avoid the oxidative damage induced by adverse conditions. In this study, plants treated with FA showed higher SOD, CAT, POX, and APX activity than untreated plants under sodic-alkaline stress. Several previous studies have confirmed that exogenous FA can enhance the activities of antioxidant enzymes under adverse-stress conditions [67,71,72]. These impacts could maintain the steady state of ROS in plant tissues leading to enhancement of signaling transduction, cell division, and the performance of different physiological and developmental processes [15,20,21,23]. On the other hand, except for POX, no significant differences were detected between FA-treated and non-treated plants. Generally, peroxidases play a pivotal role in the development of plants. They can be involved in the biosynthesis of lignin and the formation of cell walls and xylem vessels [73]. These findings imply that FA, by its effects on POX, can be considered a powerful stimulator to cell division and plant growth under favorable and alkaline-stress conditions.

Leaf relative water content (RWC) and leaf osmoprotectant compounds are closely linked to plant tolerance to osmotic stresses, i.e., drought and salinity stress. In this study, seedlings treated with FA exhibited significant enhancement in RWC compared to untreated plants. This response could be attributed to the stabilization of the cellular membranes and maintenance of cell turgor as a result of reducing the oxidative damage to the membranes’ lipid and protein components in the FA-treated plants [8,14,20]. Moreover, there was a significant increase in the osmoprotectant compounds including FAA, proline, and soluble sugars. Folic acid and its derivatives (folates) contain glutamate residues which are involved in the biosynthesis of several amino acids such as proline, glycine, tryptophan, methionine, and valine [15]. Folates are responsible for the regulation of carbohydrate metabolism by interfering as essential cofactors in the one-carbon transfer reactions [74]. Additionally, folates can enhance the photosynthetic capacity of plants by affecting the concentration of leaf pigments [15,75]. These responses could explain the positive effect of FA on improving the concentration of soluble sugars under the circumstances of this study.

Uptake of nutrients under saline and/or alkaline stress is always correlated with the integrity of cell membranes and the selective permeability to some nutrients and not others. In this study, FA-treated plants showed a significant increase in the uptake of K and Ca, while Na and the Na/K ratio were significantly decreased under sodic-alkaline stress compared to the untreated seedlings. These findings imply that FA can rectify the functions of root cells under sodic-alkaline stress leading to improvement in their ability to uptake K and Ca in parallel with reducing Na uptake. Generally, use of compounds that have strong antioxidative properties can protect the stability of the cell membrane and achieve appropriate selective permeability to various nutrients [10,50]. On the other hand, it was obvious that there was an improvement in K and Ca uptake in the FA-treated seedlings under non-alkaline conditions compared to the untreated seedlings. These results could be attributed to enhancement of the plant–water relations and nutrient transport via xylem tissue [5].

In the present work, molecular studies using RT-qPCR demonstrated that sodic-alkaline stress significantly down-regulated the relative expression of *D2-Protein* and *PEP-case* compared to the unstressed seedlings. These findings imply that maize seedlings, as a C4 plant, tended to decrease their photosynthetic capacity under sodic-alkaline stress through affecting the major core protein of photosystem II (*D2-Protein*) and the activity of the first enzyme of carbon fixation cycle in C4 plants (*PEP-case*). This strategy can enable plants to survive by reducing the excessive release of ROS from chloroplasts under sodic-alkaline stress. Meanwhile, the up-regulation of *ZmSOS1* and *ZmNHX1* and the opposite trend of *ZmHKT1* can also enable the stressed plants to survive by excluding Na^+^ from the cytosol to apoplast or vacuole, respectively, leading to protection of the cytosolic enzymes from the toxicity of Na ions. Conversely, applied FA led to up-regulation of the expression of *ZmHKT1*, *D2-Protein,* and *PEP-case* and down-regulation the expression of *ZmSOS1* and *ZmNHX1.* These responses indicated that FA has the ability to enhance the K^+^/Na^+^ selectivity and the photosynthetic capacity of maize plants under sodic-alkaline conditions. In this study, these effects were previously confirmed by enhancing the concentration of photosynthetic pigments (Figure 1) and K^+^/Na^+^ ratio (Figure 5).

## 5. Conclusions

**Figure 7:**
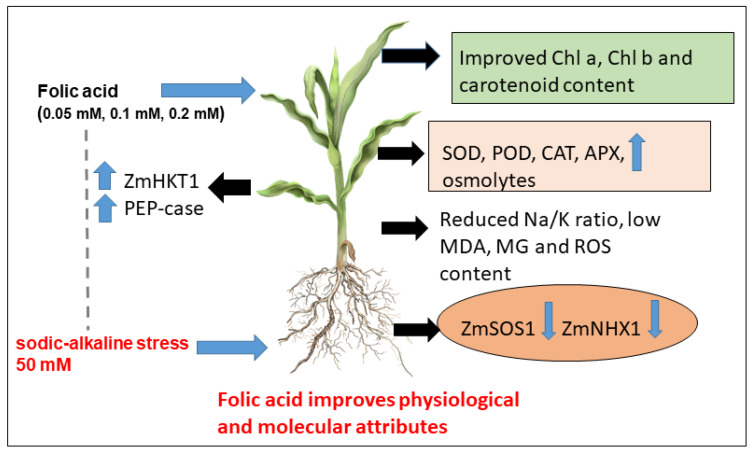
Summarized events of folic-acid-mediated sodic-alkaline stress tolerance in maize seedlings (Abbreviations: ZmHKT1, high-affinity potassium transporter protein; PEP-case, phospheonol pyruvate carboxylase; SOD, superoxide dismutase; POD, peroxidase; CAT, catalase; APX, ascorbate peroxidase; MG, methyglyoxal; MDA, malondialdehyde; ROS, reactive oxygen species; Zm NHX1, sodium/hydrogen exchanger 1; ZmSOS1, salt overly sensitive 1.

The present work provides evidence of the ameliorative role of folic acid (foliar spray) for sodic-alkaline-stressed maize seedlings. The various physiological and molecular attributes examined demonstrated that folic acid up-regulated the antioxidative defense, pigment accumulation, photosynthetic efficiency, and carbon fixation in alkaline-stressed maize seedlings (Figure 7). Furthermore, in view of sodicity as a stress combined with alkalinity, folic acid supplementation revealed unique mechanisms of ion homeostasis manifested by altered gene expression of K^+^ channels and Na^+^/H^+^ antiport. This indicates that folic acid brings about restriction of Na^+^ accumulation accompanied by its vacuolar sequestration in the leaves of maize seedlings subjected to sodic-alkaline stress. The plants supplemented with folic acid also exhibited better osmotic tolerance evident from improved RWC content and lower accumulation of MDA and MG. Moreover, FAA, proline, and sugar contents were also improved in the presence of folic acid. Further investigations are required to decipher the mechanisms of folic-acid-mediated tolerance in maize seedlings, and detailed objectives are required to be accomplished for the biosynthetic pathways of each of the parameters. Moreover, it is imperative to understand the molecular mechanisms of crosstalk between folic acid and other stress-related signaling molecules such as NO, H_2_S, and phytohormones, to enable us to decipher the mode of action of folic acid in multiple routes. Lastly, the present work has potential agronomic importance for folic acid application in the management of crops in extensively fertilized (alkaline) or salinity-affected (sodic) agricultural soils in arid regions world-wide. Maize is an important staple cereal which needs sustainability in diverse agro-climatic regions with varied soil profiles. Folic acid can also be provided after detailed physiological and molecular investigations.

## Figures and Tables

**Table 1:** List of primers.

Primer Name		Sequence	Tm
ZmHKT1	F	5′-TGCTAATGTTTATCGTGCTG-3′	56 °C
	R	5′-AGGCTGATCCTCTTCCTAAC-3′	
ZmSOS1	F	5′-ACTTGCAGGAGGAATACAAC-3′	
	R	5′- CGAGAAGAGAAGACCACATC-3′	
ZmNHX	F	5′-CGTGATGTCGCATTACACCT-3′	
	R	5′-CTGGCAAACTCCCACTTCTC-3′	
D2 protein	F	5′-GGAAGATCAATCGACCGAAA-3′	
	R	5′-CCTTATGCACCCATTTCACA-3′	
PEP case	F	5′-AGCCTTCAGAACCGATGAAATC-3′	
	R	5′-CATCCCATAGCGCATTTCG-3′	
β-Actin	F	5’-GTGCCCATTTACGAAGGATA-3′	
	R	5′-GAAGACTCCATGCCGATCAT-3′	

## Data Availability

Not applicable.
